# Recent Uptrend in Whole-Grain Intake Is Absent for Low-Income Adolescents, National Health and Nutrition Examination Survey, 2005–2012

**DOI:** 10.5888/pcd14.160540

**Published:** 2017-07-06

**Authors:** June M. Tester, Cindy W. Leung, Tashara M. Leak, Barbara A. Laraia

**Affiliations:** 1 UCSF Benioff Children’s Hospital Oakland, Oakland, California; 2 Center for Health & Community, University of California, San Francisco, San Francisco, California; 3 School of Public Health, University of California, Berkeley, Berkeley, California

## Abstract

**Introduction:**

Whole-grain consumption reduces risk of chronic disease, yet adolescents consume suboptimal amounts. It is unclear whether trends in consumption of whole grains have been positive among adolescents, and research assessing disparities by socioeconomic status is limited. The objective of our study was to evaluate recent trends in whole-grain consumption by US adolescents.

**Methods:**

We examined data on 3,265 adolescents aged 13 to18 years from the National Health and Nutrition Examination Survey (NHANES) 2005–2012. Intake of whole and refined grains was analyzed by using generalized linear models, and odds of no whole-grain intake were examined with logistic regression, adjusting for socioeconomic and demographic factors. We evaluated trends and examined heterogeneity of trends with respect to annual household income.

**Results:**

Daily whole-grain consumption among adolescents increased overall by about a quarter-ounce–equivalent per day (oz-eq/d) (*P* trend <.001). We found a significant relationship between whole-grain intake and income. Daily whole grains (recommended as ≥3 oz-eq/d), increased (0.6 to 1.0 oz-eq/d) among high-income adolescents (*P* trend < .001) but remained at 0.5 oz-eq/d for low-income adolescents. The ratio of whole grains to total grains (recommended to be at least 50%) rose from 7.6% to 14.2% for high-income adolescents (*P *trend < .001), with no significant trend for the low-income group. Consumption of refined grains did not change. Odds of having no whole grains trended downward, but only for the high-income adolescents (*P* trend = .01).

**Conclusion:**

These data show significant (albeit modest) trends toward increased intake of whole grains among high-income adolescents nationwide that are absent among low-income peers. Future interventions and policies should address barriers to whole-grain consumption among this vulnerable group.

## Introduction

Whole-grain consumption among adolescents has been associated not only with weight status but with insulin resistance; high whole-grain consumption is linked to reduced levels of diabetes risk indicators such as fasting insulin and C-peptide (1) as well as to directly measured insulin sensitivity (using euglycemic clamp technique) (2). However, despite these health benefits, consumption of whole grains among adolescents has long fallen short in the United States (3). Whole grains are raw, cooked, or produced foods that are made with 100% of the grain kernel that includes the bran, germ, and endosperm (4). Examples of whole grains include whole wheat, amaranth, barley, buckwheat, corn, millet, oats, quinoa, brown rice, rye, sorghum, teff, triticale, and wild rice. Since 2005, the Dietary Guidelines for Americans have included a specific recommendation that at least half of total grain intake come from whole grains. The recommended amount of grains at the 2,000-calorie level is 6 ounce-equivalents (oz-eq) per day, of which at least 3 ounce equivalents should be whole grains(5)(6).

National studies evaluated patterns in consumption of whole grains among children and adolescents. All point to low levels of consumption (3,7,8), with a typical intake of about a half-ounce serving equivalent per day of whole grains (ie, half a slice of whole wheat bread) among 6 to 18 year olds in 2001–2002 (7). Some studies suggest a modest increase during the past 10 years (7), whereas others suggest no change (8). These reported trends were measured among adolescents together with young children, whose diet quality is known to be more favorable than that of adolescents (9). A regional study of teenagers showed that from 1999 through 2004, daily intake of whole grains was slightly more than a half-serving per day (10). This study also found that whole-grain intake was lower among adolescents of high socioeconomic status (10), which was somewhat counterintuitive given the evidence that low-income children and adolescents generally have poorer diets than their wealthier peers (11). Information is needed on national trends for adolescents since the updated dietary guidelines in 2005, trends that also take into account socioeconomic status (SES). To learn how SES affects trends in whole-grain consumption over time, we examined trends in whole-grain intake by using 24-hour dietary recall data from adolescents in the National Health and Examination Survey (NHANES) from 2005 through 2012. 

## Methods

### Study population

We analyzed data on participants in NHANES, which is a multistage probability cross-sectional sample designed to be representative of the noninstitutionalized US civilian population. This analysis combined data from 2005, through 2012 (ie, 4 two-year data cycles including 2005–2006, 2007–2008, 2009–2010, and 2011–2012). The data were analyzed in 2016.

The NHANES Household Questionnaire yielded 3,816 adolescents aged 13 through 18 years. Of these, 3,265 had complete demographic information (sex, race, age, education and marital status of household respondent, food security), anthropometric measurements (height and weight), and 2 days of diet recall; the final sample consisted of these 3,265 adolescents.

### Measures

Anthropometric measurements were made during the in-person examination at the mobile examination center per protocol (12). Demographic information collected was the adolescent’s race/ethnicity, age, sex, annual household income, household food security status, and household respondent’s partnered status (partnered or married versus single or unpartnered) as well as educational attainment (less than high school diploma and no general equivalency degree versus high school diploma or more). Questions about the household such as food security status and family income were asked of a caregiver or parent. Data on annual household income and family size were used with each cycle of NHANES to calculate the index of family income to the federal poverty level (FPL) in accordance with the US Department of Health and Human Services’ poverty guidelines (13). For this analysis, household income at or below 200% of the FPL was categorized as low income and income above 200% of FPL was categorized as high income.

A 24-hour diet recall was conducted in person at the mobile examination center and a second recall was collected via telephone. According to protocol, diet recalls were conducted directly with the adolescent (14). The Food Pattern Equivalents Database (FPED) was used to convert foods and beverages reported in NHANES to US Department of Agriculture food pattern components (15). The FPED database reports grain consumption in ounce-equivalents to align with the food group serving definitions used in the 2005 Dietary Guidelines for Americans. Examples of an ounce-equivalent include the following: one slice of bread, one cup of cereal, or a half cup of hot cereal, cooked pasta, rice, or other grain such as bulgur, oatmeal, or cornmeal (15). The average of the 2 recalls was used for this analysis.

The diet outcome of interest was consumption of whole grains, reported in the FPED database as ounce-equivalents of whole grains. The mean intake of whole grains and refined grains was analyzed. The proportion of total grains consumed that were whole grain was also examined and was calculated as the ratio of ounce-equivalents of whole grains divided by the sum of whole grains and refined grains. Adolescents were also dichotomized as having no whole grains if the average whole-grain consumption of their 2 diet recalls was 0, and as having some whole grains if the average was any number greater than 0.

### Statistical analyses

Survey weights were used to account for the complex, multistage probability sampling design used in NHANES in accordance with recommendations from the National Center for Health Statistics (13). Standard errors were estimated using jackknife replication. Analyses were conducted using Stata 12.1 (StataCorp LLC).

For descriptive analyses, low-income adolescents were compared with high-income adolescents by using design-based χ^2^ tests for categorical variables (eg, sex) and by using linear regression for continuous variables (eg, age).

We compared low-income groups to high-income groups in the baseline (2005–2006) and final (2011–2012) cycles. We conducted a basic comparison of group means (whole grains, refined grains, percentage with whole grains, percentage with no whole grains) between income groups; unadjusted linear regression was conducted for each whole-grain outcome with a dichotomous variable (high-income versus low-income). Conducting a simple unadjusted regression in this manner is equivalent to performing a *t* test of means while using survey-weighted data. 

For trend analysis, whole grains, refined grains, and percentage of whole grains were skewed to the right (positive skew) and were analyzed by using generalized linear model assuming a γ distribution with a log link. Survey year was included as an ordinal term, and significance of a linear trend was tested with post-estimation by using contrast coefficients for survey year as a 4-level predictor (−β_2_ +β_3_ + 3β_4_ = 0) (16). Logistic regression was conducted to analyze the odds of consuming any whole grains on the 2 days surveyed, and significance of a linear trend was similarly tested with post-estimation by using the same contrast coefficients for survey year. All models were adjusted for the following covariates: age, race/ethnicity, sex, weight status (normal [body mass index (BMI) <85th percentile], overweight [85th–94th percentile], or obese [≥95th percentile]) and average total energy intake of the child; education and marital status of the household respondent; household income-to-poverty ratio and household food security status. *P* < .05 was the statistical threshold for significance.

We tested for an interaction between income group (low-income and high-income) and survey year for each outcome of interest by performing post-estimation with a Wald test, using *P* < .20 as a relaxed threshold that accepts a higher type I error rate when assessing interactions (17).

## Results

Adolescents in the high-income and low-income groups did not differ from one another with respect to sex or age when using *P* < .05 as a threshold for significance (Table 1). We found significant differences in race/ethnicity, weight status, food security status, educational attainment, and partnership status (*P* < .01 for all), and a higher proportion of low-income adolescents (compared with high-income peers) were Hispanic or non-Hispanic black, overweight/obese rather than normal weight, living with household food insecurity, and from a household whose parent/caregiver had less than a high school education and was single/unmarried. Total energy intake was lower among the adolescents in the low income group (*P* < .001).

**Table 1:** Demographic Characteristics of Study Sample of Adolescents Aged 13 to 18 Years (N = 3,265), National Health and Nutrition Examination Survey 2005–2012

Characteristic	Low Income, n = 1,740^a^	High Income, n = 1,525^a^	*P* Value
**Sex **			
Male	838 (45.6)	799 (51.8)	.05^b^
Female	902 (54.4)	726 (48.2)	
**Race/ethnicity**			
Non-Hispanic white	355 (44.2)	636 (75.8)	<.001^b^
Hispanic	717 (28.9)	364 (10.3)	
Non-Hispanic black	556 (19.9)	379 (9.2)	
Other/mixed	112 (7.0)	146(4.7)	
**Education, household respondent**			
Less than high school diploma, no GED	754 (34.4)	180 (8.5)	<.001^b^
High school diploma or more	986 (65.6)	1,345 (91.5)	
**Marriage/partner status, household respondent**			
Single/unpartnered	813 (42.6)	315(19.6)	<.001^b^
Married/partnered	927 (57.4)	1,210 (80.4)	
**Household food security^d^**			
Food secure	789 (48.8)	1,296 (87.9)	<.001^b^
Marginally food secure	292 (14.4)	105 (5.6)	
Food insecure	659 (36.8)	124 (6.5)	
**Weight^d^**			
Normal	1,024 (62.7)	1,024 (70.0)	.004^b^
Overweight	287 (15.0)	239 (15.1)	
Obese	411 (22.3)	262 (14.9)	
**Age, mean (SE), y**	15.4 (0.07)	15.4 (0.08)	.79^c^
**Annual household income, mean (SE), % FPL**	104 (2.4)	382 (4.9)	<.001^c^
**Total daily energy consumed, mean (SE), kcal**	1,962 (31)	2,157 (34)	<.001^c^

Abbreviations: SE, standard error; FPL, federal poverty level; GED, general equivalency degree.

a Values are n (%) unless otherwise indicated.

b *P* values were derived from design-based *F* test (χ^2^).

c *P* value is for unadjusted linear regression with each continuous variable as the outcome and income category as the lone predictor.

d Weight categories are normal (body mass index [BMI] <85th percentile), overweight (BMI 85th to 94th percentile), obese (BMI ≥95th percentile).

The interactions between survey year and income group were all below *P* = .20: whole grains (*P* = .06), refined grains (*P* = .04), percentage whole grains (*P* = .003), odds of no whole grains (*P* = .19). Testing was therefore done separately for the low-income and high-income groups.

At baseline (2005–2006) we found no significant difference between low-income and high-income groups with respect to whole grain ounce-equivalents, the percentage of grains consumed that were whole grain, or the proportion of adolescents having zero intake of whole grains (all *P* > .05). Refined grains intake was higher for the high-income group (*P* = .01).

In 2011–2012, we found no significant income-group difference in refined grains intake (*P* > .05). However in the 2011–2012 cycle, the high-income group compared with the low-income group had higher whole-grain intake (*P* = .002), consumed a higher percentage of grains that were whole grain (*P* = .008), and had a lower proportion with no whole-grain intake (*P* = .03).

Overall, consumption of whole grains increased from 0.5 ounce-equivalent per day (2005–2006) to 0.8 ounce-equivalent per day (2011–202), *P* trend <.001 (Table 2). When stratified by income group, whole-grain intake in high-income adolescents trended upward from 2005–2006 through 2011–2012, from 0.6 ounce equivalent per day (2005–2006) to 0.6 ounce equivalent per day (2007–2008), 0.7 ounce equivalent per day (2009–2010), and 1.0 ounce equivalent per day (2011–2012) (*P* trend < .001). In 2005–2006, low-income adolescents consumed an average of 0.5 ounce equivalents per day of whole grains, and in subsequent cycles, this finding remained comparable at 0.5 (2007–2008), 0.6 (2008–2009), and 0.5 ounce equivalents per day (2011–2012) (*P *trend 0.08) (Table 2). We found no significant trends in refined grain intake for either income group.

**Table 2:** Trends in Whole-Grain and Refined-Grain Intake Among Adolescents Aged13 to 18 years, National Health and Nutrition Examination Survey, 2005–2012

Income Category	Grain Intake	2005–2006	2007–2008	2009–2010	2011–2012	*P* Value of Linear Trend^a^
Low income^b^	No. surveyed	646	321	391	382	NA
	Whole grains, oz	0.5	0.5	0.6	0.5	.08^c^
	Refined grains, oz	6.4	5.7	6.2	6.3	.06^c^
	Whole grain/total grains, %	6.8	9.8	8.2	9.0	.14^d^
	No whole grains, %	41.7	31.1	37.3	34.0	.36^d^
High income^b^	No. surveyed	634	301	301	289	NA
	Whole grains, oz	0.6	0.6	0.7	1.0	<.001^c^
	Refined grains, oz	7.1	6.4	7.3	6.0	.19^c^
	Whole grain/total grains, %	7.6	9.2	9.5	14.2	<.001^d^
	No whole grains, %	34.9	33.1	29.2	21.7	.01^d^
All incomes	No. surveyed	1,280	622	692	671	NA
	Whole grains, oz	0.5	0.6	0.6	0.8	<.001^c^
	Refined grains, oz	6.8	6.1	6.8	6.1	.92^c^
	Whole grain/total grains, %	7.3	8.9	8.8	11.9	<.001^d^
	No whole grains, %	37.3	32.3	32.7	27.2	.007^d^

Abbreviation: NA, not applicable.

a All models were adjusted for the following covariates: age, race/ethnicity, sex, weight status (normal [body mass index (BMI) <85th percentile], overweight [BMI 85th–94th percentile], or obese [BMI ≥95th percentile], and average total daily energy intake of the child; education and marital status of the household respondent; household income-to-poverty ratio; and household food security status.

b Household income at or below 200% of the federal poverty level (FPL) was categorized as low income; income above 200% of FPL was categorized as high income.

c Significance of linear trend done by conducting generalized linear regression model for respective whole-grain outcomes; *P* value from post-estimation using contrast coefficients for survey year as an ordinal term.

d Significance of linear trend calculated by conducting logistic regression of odds of no whole grains; *P* value from post-estimation calculated by using contrast coefficients for survey year as an ordinal term.

From 2005–2006 through 2011–2012, the overall percentage of grains that were whole grains consumed by adolescents rose significantly from 7.3% to 11.9% (*P* trend < .001) but was below the recommended level of at least 50%.

In 2005–2006, the percentage of total grains consumed that were whole grains was 6.8% among low-income adolescents, and the percentages in subsequent cycles were 9.8% (2007–2008), 8.2% (2009–2010), and 9.0% (2011–2012). Test of linear trend was not significant (*P* trend = .14). The percentage of grains consumed that were whole grains was 7.6% among high-income adolescents at baseline, and in subsequent cycles, the percentage increased to 9.2% (2007–2008), 9.5% (2009–2010), and 14.2% (2011–2012) (*P* trend = < .001).

In 2005–2006, more than 1 in 3 adolescents (41.7% low-income and 34.9% high-income) consumed no whole grains on either of the 2 days they were surveyed (Table 2). Overall, there was a significant overall linear trend toward decreasing odds of having no intake of whole grains (*P* trend = .007), and by 2011–2012, this proportion was closer to 1 in 4 adolescents. When stratified by income group, the proportion of high-income adolescents with no whole-grain intake decreased over time: 34.9% (2005–2006), 33.1% (2007–2008), 29.2% (2009–2010), and 21.7% (2011–2012) (*P* trend = .01). However, there was no evidence of a significant trend for low-income adolescents: 41.7% (2005–2006), 31.1% (2007–2008), 37.3% (2009–2010), and 34.0% (2011–2012) (*P* trend = .36).

## Discussion

This study examined mean adjusted trends in whole-grain consumption by US adolescents aged 13 to 18 years. In general, adolescents showed some increases in consumption, but continued to have inadequate whole-grain intake. Overall, daily consumption of whole grains is still no more than one-third of the lowest recommended amount, only one-eighth (rather than one-half) of consumed grains are whole grains, and on average about 1 in 4 adolescents still does not eat any whole grains at all on a typical day. Many factors could explain why, irrespective of socioeconomic factors, adolescents are not consuming recommended amounts of whole grains. Many households in the United States may be unfamiliar with how to cook whole grains (eg, quinoa) (18). Decreased preference because of the taste and texture of whole grain foods also is an issue (19).

This study shows a modest but consistent trend toward increased whole-grain consumption only among high-income adolescents. A recent analysis of trends in dietary intake among adults (1999–2012) showed that although overall disparities in self-reported diet either persisted or worsened, consumption of whole grains roughly doubled for adults of all SES levels (20). Research is needed to explore why low-income adolescents are not increasing their consumption of whole grains.

Recent policies aimed to increase the availability of whole grains for children. The Healthy Hunger-Free Kids Act of 2010 brought key improvements to school lunches, such as including more foods rich in whole grains, and there is evidence of improved availability of whole grains in elementary (21) and secondary schools (22). In 2009 wide-ranging changes to the Special Supplemental Nutrition Program for Women, Infants and Children (WIC) Program included an emphasis on whole-grain foods such as whole-grain bread and brown rice. Evidence shows improved whole-grain intake among low-income children younger than 5 years (23). However, no similar policies are aimed directly at adolescents in low-income households.

Although high-income adolescents increased their whole-grain intake, this increase was not accompanied by a decrease in refined grains. This finding suggests that adolescents may not be substituting whole grains for refined grains so much as adding whole-grain products to their diet. There is value in creative strategies from industry for increasing the whole-grain content of commonly eaten foods such as breakfast cereals and also improving the overall appeal of whole-grain foods to the public. Analytic modeling shows that even modest substitutions of whole-grain flour for foods commonly eaten by children and adolescents could result in meaningful increases in whole-grain intake at a population level (24), and interventions evaluated the acceptability of substituting whole grains for refined grains in foods such as pizza crust (25).

Given the disproportionate burden of obesity and chronic disease on low-income youths, this widening gap among adolescents is cause for concern. It is possible that regardless of interest or demand for whole-grain products, the higher price of whole-grain foods (26) prevents low-income households from purchasing or consuming these foods. Caregivers in low-income households are less tolerant of taking a risk on purchasing a food that might be rejected (eg, because of unfamiliarity or taste preference) for fear of spoilage and waste of financial resources (27). If cost is indeed the most prohibitive barrier, policies addressing the higher cost of whole-grain products will be crucial for meaningful population change.

This study’s analysis has limitations. Because of the cross-sectional nature of NHANES, inferences of causality cannot be made. Dietary intake was assessed by using the average of two 24-hour diet recalls, which relied on self-report. Parents and children have difficulty recognizing whole-grain foods (28), and even food service personnel struggle with reading labels to determine the whole-grain content (29). Furthermore, there are limitations to using 24-hour diet recalls to model usual intake of foods in a population when these foods are only consumed episodically (30). The study sample included a wide age range of adolescents without available information about pubertal stage, which is a potential limitation given the behavioral and biologic differences among adolescents of various ages.

The findings in this study illustrate overall trends in whole-grain intake, highlighting the influence of income level on this intake. Future analyses should explore in greater depth potential patterns in the types of whole-grain foods that are being consumed more frequently by high-income adolescents.

Eight years after the Dietary Guidelines for Americans introduced more specific recommendations about the quantity of whole grains that should be consumed , daily consumption of whole grains among adolescents remains low. Therefore interventions and policies should focus on increasing whole-grain consumption among adolescents. In particular, additional attention should be focused on low-income adolescents who may have more barriers to whole-grain consumption and who are therefore at higher risk for diet-related chronic disease than high-income adolescents.
